# A Randomized Controlled Trial of Novel Treatment for Hemorrhagic Radiation Proctitis

**DOI:** 10.31557/APJCP.2020.21.10.2927

**Published:** 2020-10

**Authors:** Wei Chieng Pui, Tiong How Chieng, Sze Li Siow, Nik Azim Nik Abdullah, Ismail Sagap

**Affiliations:** 1 *Department of Surgery, Sibu Hospital, Sarawak, Malaysia. *; 2 *Department of Surgery, Sarawak General Hospital(SGH), Sarawak, Malaysia. *; 3 *Colorectal Unit, Department of Surgery, Universiti Kebangsaan Malaysia(UKM), Kuala Lumpur, Malaysia. *

**Keywords:** Hemorrhagic radiation proctitis, water irrigation, oral antibiotics, formalin, per rectal bleeding

## Abstract

**Background:**

Various methods have been used for treatment of hemorrhagic radiation proctitis (HRP) with variable results. Currently, the preferred treatment is formalin application or endoscopic therapy with argon plasma coagulation. Recently, a novel therapy with colonic water irrigation and oral antibiotics showed promising results and more effective compared to 4% formalin application for HRP. The study objective is to compare the effect of water irrigation and oral antibiotics versus 4% formalin application in improving per rectal bleeding due to HRP and related symptoms such as diarrhoea, tenesmus, stool frequency, stool urgency and endoscopic findings.

**Methods:**

We conducted a study on 34 patients with HRP and randomly assigned the patients to two treatment arm groups (n=17). The formalin group underwent 4% formalin dab and another session 4 weeks later. The irrigation group self-administered daily rectal irrigation at home for 8 weeks and consumed oral metronidazole and ciprofloxacin during the first one week. We measured the patients’ symptoms and endoscopic findings before and after total of 8 weeks of treatment in both groups.

**Results:**

Our study showed that HRP patients had reduced per rectal bleeding (p = 0.003) in formalin group, whereas irrigation group showed reduced diarrhoea (p=0.018) and tenesmus (p=0.024) symptoms. The comparison between the two treatment arms showed that irrigation technique was better than formalin technique for tenesmus (p=0.043) symptom only.

**Conclusion:**

This novel treatment showed benefit in treating HRP. It could be a new treatment option which is safe and conveniently self-administered at home or used as a combination with other therapies to improve the treatment outcome for HRP.

## Introduction

Radiation injury has increased over the years as radiotherapy is widely used in current curative treatment for pelvic malignancies like prostate, urinary bladder, cervix, vagina, uterus and rectum. Rectum, due to its fixed position and close relation to the radiated organ, is the most frequently affected gastrointestinal site of radiation injury (Ismail and Qureshi, 2002). 

 Acute radiation proctitis occurs during or within 3 months of radiation due to direct mucosal damage. It affects 50 – 70% of post radiotherapy patients with symptoms like abdominal cramps, rectal bleeding, mucus discharge, tenesmus or diarrhea which usually resolves spontaneously. On the other hand, chronic radiation proctitis is a continuation after acute phase or after a latent period. It presents 3 months after completion of radiation with median onset at approximately 1 year. This is caused by progressive epithelial atrophy and fibrosis associated with obliterative endarteritis and chronic mucosal ischemia (Caroline, 2010). It occurs in 5 – 20% of patients post pelvic radiotherapy (Cho et al., 1995; Schultheiss et al., 1997). Patients usually present with per rectal bleeding termed as hemorrhagic radiation proctitis; fistula or stricture. Factors that increased the risk of radiation injury include the volume of tissue irradiated, dosage and type of radiotherapy, previous surgery, concomitant chemotherapy, smoking, genetic susceptibility and medical conditions like diabetes, hypertension, HIV and inflammatory bowel disease (Caroline, 2010).

Treatment modalities of hemorrhagic radiation proctitis involve medical, endoscopic and surgical therapies. Current medications used include steroid enema, sulcrafate enema, 5-ASA enema, short chain fatty acids and antioxidants such as Vitamin E (Hortelano et al., 2014). Endoscopic therapy include argon plasma coagulation (APC), formalin therapy, neodymium:Yttrium-aluminium-garnet (Nd:YAG), radiofrequency ablation or cryoablation (Hortelano et al., 2014; Karamanolis et al., 2013). Another treatment option is hyperbaric oxygen therapy (Caroline, 2010). When all methods fail, surgical therapy like diversion colostomy or proctectomy can be considered.

Endoscopic treatment has shown promising results for hemorrhagic radiation proctitis treatment (Hortelano et al., 2014; Karamanolis et al., 2013). A retrospective study comparing APC and formalin application showed that APC was more effective in controlling hematochezia and safer (Alfadhli et al., 2008). However, it was a nonrandomized trial with possibility of selection bias and no proper bleeding severity score was used as the end point assessed was normalization of hemoglobin or 10% improvement of hemoglobin from baseline. Furthermore, there was no pretreatment endoscopy to assess the severity of proctopathy or to rule out any other causes of hematochezia. A more recent randomized controlled trial showed both treatment modalities had same effect in controlling rectal bleeding but did not improve anorectal dysfunction (Yeoh et al., 2013). Some authors suggested that APC is the preferred method for mild to moderate chronic radiation proctitis and often needing a few sessions to arrest the rectal bleeding (Karamanolis et al., 2013). Contrarily, intra rectal formalin application is useful even for severe or resistant radiation proctitis (Karamanolis et al., 2013; Leiper and Morris, 2007). It is also commonly used because of its efficacy and ease of treatment. So, endoscopic treatment of choice depends on the severity of the proctitis, availability of treatment and the endoscopist’s experience in using both the treatment safely. In our setting, both APC and formalin application are used to treat this condition.

Formalin consists of methanol and formaldehyde that binds to proteins and causes cell necrosis. Thus, it causes chemical cauterization to stop bleeding from the telangiectasias in the rectal mucosa and submucosal vessels (Denton et al., 2002). Most studies used 4% formalin application using formalin-soaked gauze to dab the mucosa or by instilling the formalin solution via Foleys catheter or through the operating channel of a colonoscope (Ismail and Qureshi, 2002; Karamanolis et al., 2013; Lee et al., 2007; Gautam et al., 2010). This would be followed by water or saline irrigation to wash the formalin away. Different mucosal contact time and volumes of formalin used were also reported (Karamanolis et al, 2013). The success of homeostasis from multiple sessions’ therapy ranges from 60% to 100% (Ismail and Qureshi, 2002; Karamanolis et al., 2013; Lee et al., 2007; Gautam et al., 2010). Median follow ups until two years showed minimal relapse and complications such as anal stenosis, fissures, fecal incontinence and ulceration (Ismail and Qureshi, 2002; Karamanolis et al., 2013; Lee et al., 2007; Gautam et al., 2010). All these studies showed that the application of 4% concentration of formalin for about 3 minutes is effective in treating hemorrhagic radiation proctitis and safe with minimal complication and toxic effect.

A promising new therapy for hemorrhagic radiation proctitis using colonic irrigation with oral antibiotic administration was reported in 2011 (Sahakitrungruang et al., 2011). The patients self-administered colonic irrigation daily with 1,000ml of tap water and were given 1 – week course of oral ciprofloxacin and metronidazole. It showed significant improvement in rectal bleeding, bowel frequency, bowel urgency and diarrhea. The same authors conducted a randomized controlled trial comparing this therapy versus 4% formalin-soaked gauze application for 3 minutes showed superiority of the irrigation group in improving rectal bleeding, bowel frequency, bowel urgency, diarrhea and tenesmus after 8 weeks of treatment (Sahakitrungruang et al., 2012). This new treatment can be easily self-administered at home, more cost effective and also scores a higher patient satisfaction score compared to formalin therapy. 

The bleeding occurred due to mechanical trauma to the fragile superficial telangiectasia at the irradiated rectal mucosa from mpassage of stool (Sahakitrungruang et al., 2012; Trott et al., 1986). Hence, water irrigation helps to dilute the stool and smoothen the stool passage. They believed that irrigation reduces bacterial load at the same time. The reason to combine antibiotic basically metronidazole was to precipitate mucosal erythema regression and ulcer healing. Ciprofloxacin was added to cover for the possibility of superimposed infection caused by the colonic pathogens. 

In fact, a study had shown the effectiveness of metronidazole in treating rectal bleeding and diarrhoea due to chronic radiation proctitis (Cavcic et al., 2000). The author compared metronidazole in combination with steroid enema and mesalazine versus the same protocol without metronidazole. Metronidazole was used due to its immunomodulation effects and selective toxicity to anaerobic microorganisms that contribute to hypoxia to irradiated rectal tissue.

Our aim to replicate the study was to further strengthen the current evidence and prove the effectiveness of this new treatment modality. We wanted to assess its feasibility and whether the good results were reproducible in our own setting so that it can be used in our clinical practice. The objective of this study was to compare the effectiveness of this new treatment with formalin for treating hemorrhagic radiation proctitis. 

## Materials and Methods

We conducted a randomized controlled trial of water irrigation and oral antibiotics (Irrigation group) versus 4% formalin application (Formalin group). This study was registered under the Malaysian National Medical Research Register (Research ID: NMRR-15-2446-27356) and approved by the ethics committee of Universiti Kebangsaan Malaysia (UKM). All participants in this research consented to the study. Randomization to the two groups was done with blocked randomization with sealed envelope method. Block size was 4. 

**Figure 1:**
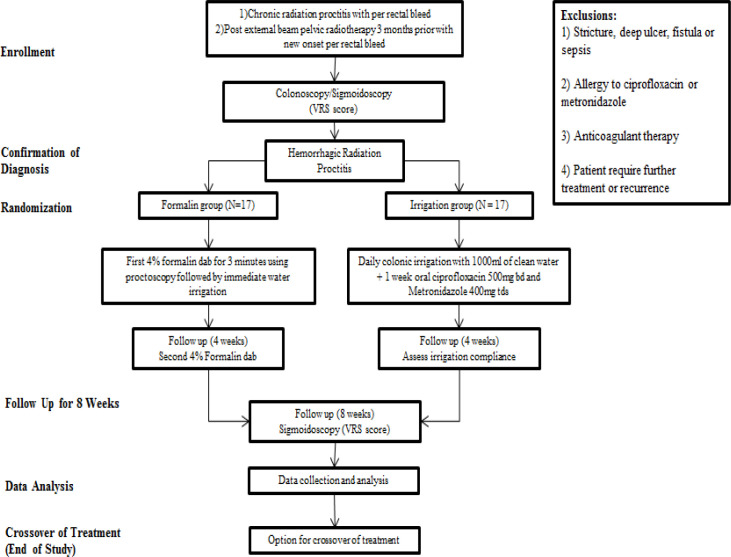
Flow Chart of Methodology - Enrollment, Randomization and Follow Up Until End of Study

This trial was conducted in Sarawak General Hospital, Kuching, Sarawak, Malaysia from 1 September 2015 until 31 May 2016. We recruited patients from outpatient clinic and inpatient admissions in the ward. This was a total 8 weeks therapy with follow up visit at 4 weeks and 8 weeks after the treatment. We did endoscopy prior treatment and at 8 weeks after treatment. Patients with first presentation of hemorrhagic radiation proctitis had full colonoscopy to rule out other causes of bleeding where as those with established radiation proctitis underwent flexible sigmoidoscopy. We evaluated the endoscopic findings using the validated Vienna Rectoscopy Score (VRS) (Wachter et al., 2000) to classify the mucosal severity of hemorrhagic radiation proctitis. We collected data on patient demographics, underlying malignancy and radiotherapy details. Patients were assessed based on per rectal bleeding (days/week), diarrhoea (days/week), tenesmus (days/week), stool frequency (times/day), stool urgency (days/week), requirement for transfusion and VRS score. Then, we randomized them into two treatment arm groups. The patients were given the option of crossover to the other group after completing 8 weeks of treatment if the bleeding did not improve or if they wished to try a different treatment. The flow chart of methodology is shown in Figure 1.

*Inclusion criteria*

Patients who previously undergone external beam pelvic radiation more than 3 months ago and has hemorrhagic radiation proctitis with at least one per rectal bleeding per week.

*Exclusion criterias*

- Patients with chronic radiation proctitis with major complications like stricture, fistula, deep ulcer and sepsis

- Patients with hemorrhagic radiation proctitis but need further surgery, chemotherapy or radiotherapy for their primary disease

- Patients allergic to ciprofloxacin and metronidazole

- Patients who are given any form of treatment like formalin, APC or steroid therapy within the period of less than 1 month

- Patients on antigoagulant

*Irrigation group*

In this group, we educated the patients on the proper irrigation technique before starting the treatment so that they fully understood all the steps needed. They self-administered rectal irrigation using 1,000 ml of clean water via a size 20F Foleys catheter. We advised them to place the catheter into the rectum with lubricant gel until just above the anorectal junction about 3 to 5 cm deep from the anal verge. The catheter was connected to a urine collection bag with cut out hole at the opposite end. Then, we instructed the patients to pour water into the urine bag via the hole and let it flow into the rectum at low gravitational pressure by holding the bag at just above the shoulder level. They would hold the irrigation water in the rectum for about 5 minutes each time. We advised them to use 500 ml drinking water bottle for easier measurement and this could be done in two sessions until the total 1,000 ml were irrigated which usually took about 15 to 20 minutes. We also suggested them to use the sitting toilet bowl for convenience or they could simply do this in the toilet with a stool on top of a squatting toilet bowl. At the same time, they consume oral ciprofloxacin 500mg twice daily and oral metronidazole 400 mg 3 times daily for the first week. Then, we followed them up on the 4^th^ week to assess symptoms and compliance to proper irrigation technique. Finally at the 8th week, we reassessed the symptoms and VRS score using sigmoidoscopy. 

*Formalin group*

We dabbed gauzes soaked with 4% formalin onto the affected rectum for 3 minutes under direct vision using proctoscopy. Then, we immediately flushed the anorectal region with about 500ml of water. This was done at the daycare operation theater. Subsequently, we followed up at the 4^th^ week to review symptoms and repeated the formalin dab. At the 8th week, we reviewed their post treatment symptoms and performed sigmoidoscopy for VRS score after the therapy.

*Statistical Analysis*

Sample size calculation was done using PS program V3.1.2 (Dupont and Plummer, 1990) by using standard deviation of 2, estimated difference in the experimental and control mean of 2 in the pilot study (Sahakitrungruang et al., 2011), power of 0.80 and type 1 error probability of 0.05. The sample size needed was 17 for each group. We estimated the dropout rate is 20% and thus, the required sample size is 22 per arm. 

We used IBM SPSS Statistics 20 for the statistical analysis. Non parametric test was used to analyze the abnormally distributed data and shown as median with interquartile range. The results of each treatment arm were evaluated using Wilcoxon signed rank test. A Mann-Whitney U test was used for the comparisons of results between the two treatment arms. 

## Results

Forty four patients were assessed during the period of this study. Nine of them were excluded as 5 had minor symptoms, 2 had deep ulcerations, one had stricture and one had fistula. The remaining total of 35 were recruited and randomized to formalin group with 17 patients and irrigation group with 18 patients respectively. However, one patient dropped out from the irrigation group and hence both groups eventually have equal 17 patients completing the study. All the patients in this study were female. 

Patient demographics were summarized in Table 1. The mean age of patients in the irrigation group was younger at 56 compared to 62 in the formalin group. There were 18 (54%) patients with cervical cancer, 15 (44%) with uterine cancer and 1 (3%) with vaginal cancer who all underwent external beam radiotherapy. The mean onset of per rectal bleeding was 11.8 months after completion of radiotherapy. The mean duration of per rectal bleeding was 14.5 months until enrollment into this study. One patient in the formalin group and 2 patients in the irrigation group had previous blood transfusion within a month period. However, none in both groups needed any blood transfusion during the study period.

Fifteen of the 17 patients in the formalin group had anorectal discomfort during the formalin dab which resolved by the following day. One patient in the irrigation group had lower colicky abdominal pain upon starting of irrigation which slowly resolved after a week and she was able to complete the 8 week treatment. No serious adverse drug reaction towards ciprofloxacin or metronidazole was reported in the irrigation group.

**Figure 2:**
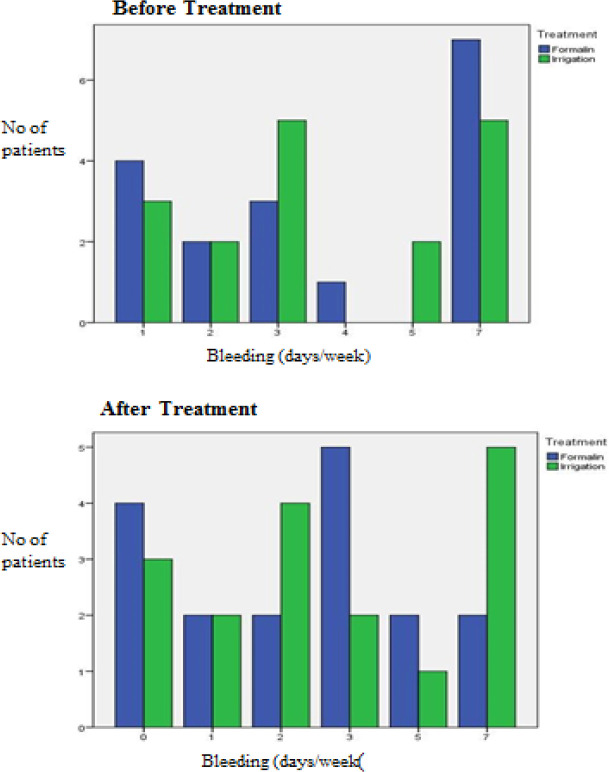
Per Rectal Bleeding before and after Treatment

**Figure 3:**
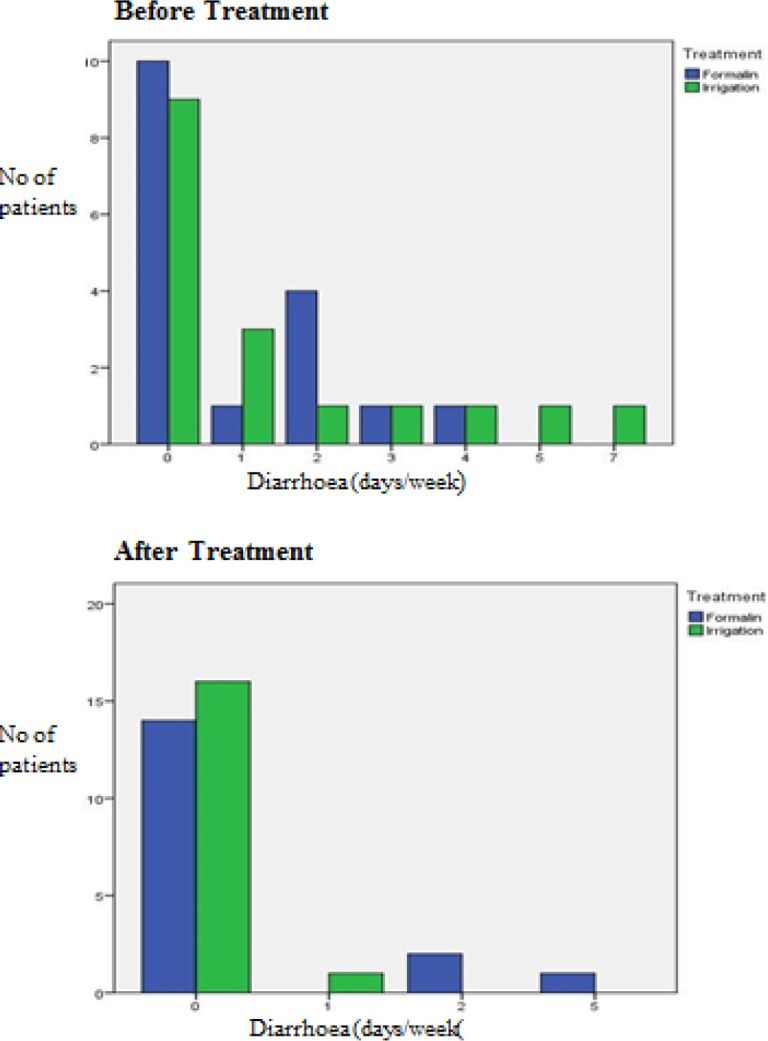
Diarrhoea before and after Treatment

**Figure 4:**
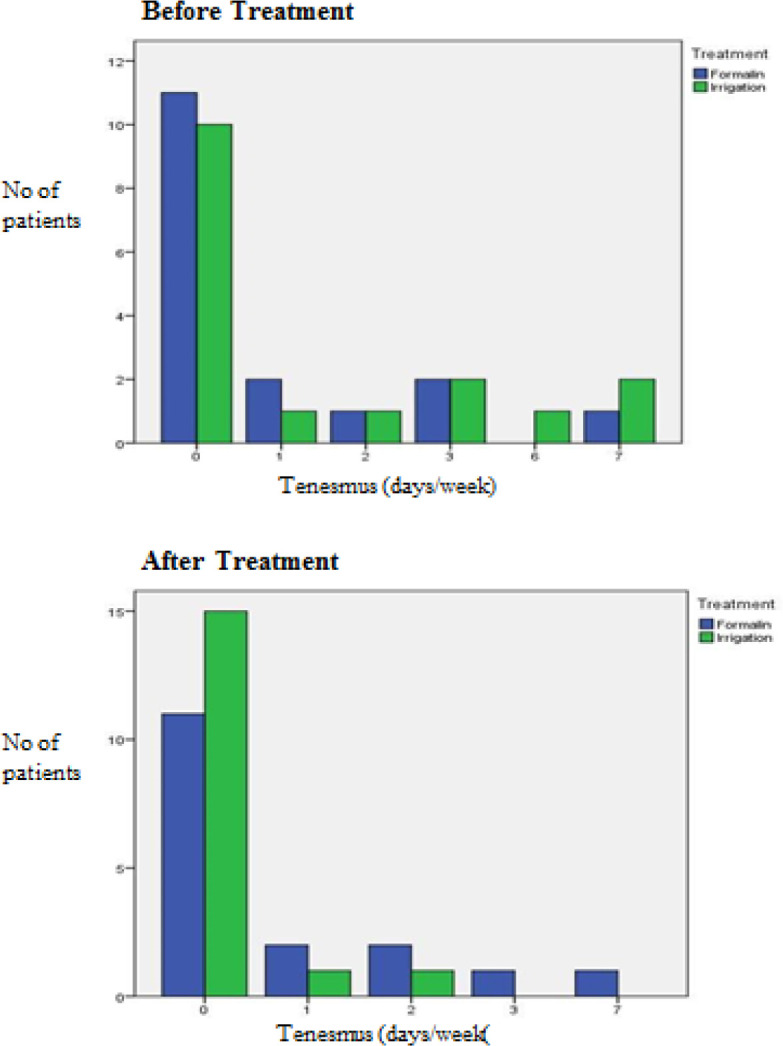
Tenesmus before and after Treatment

**Figure 5:**
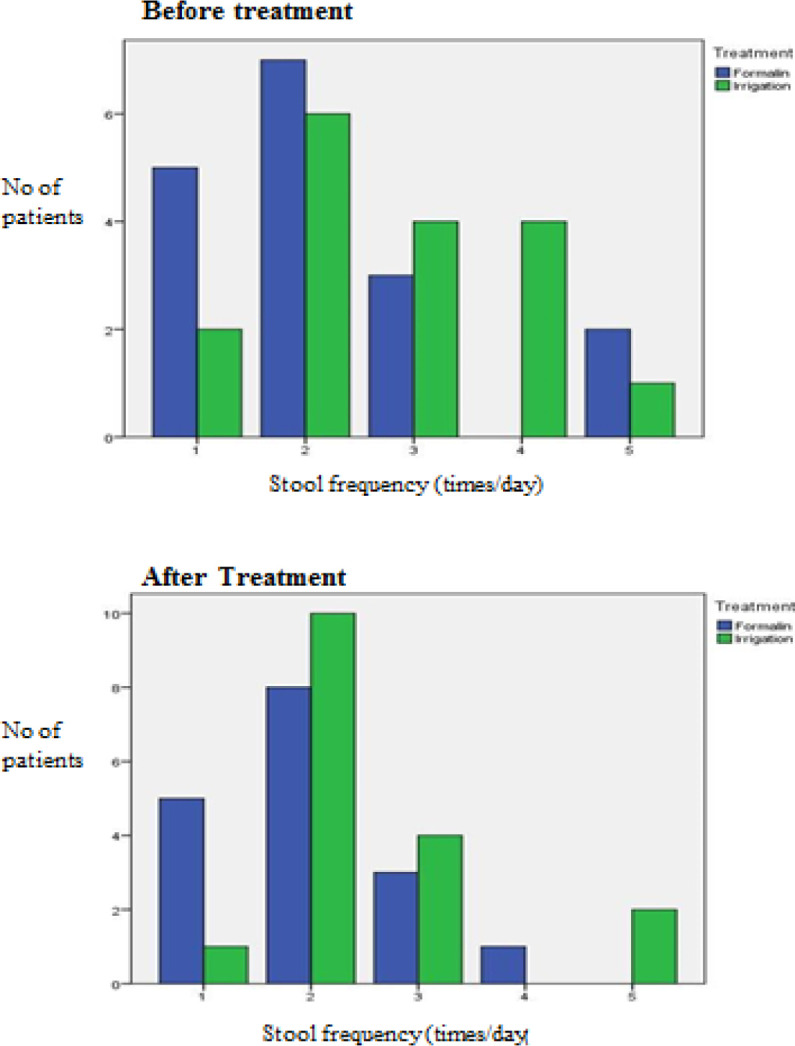
Stool Frequency before and after Treatment

**Figure 6:**
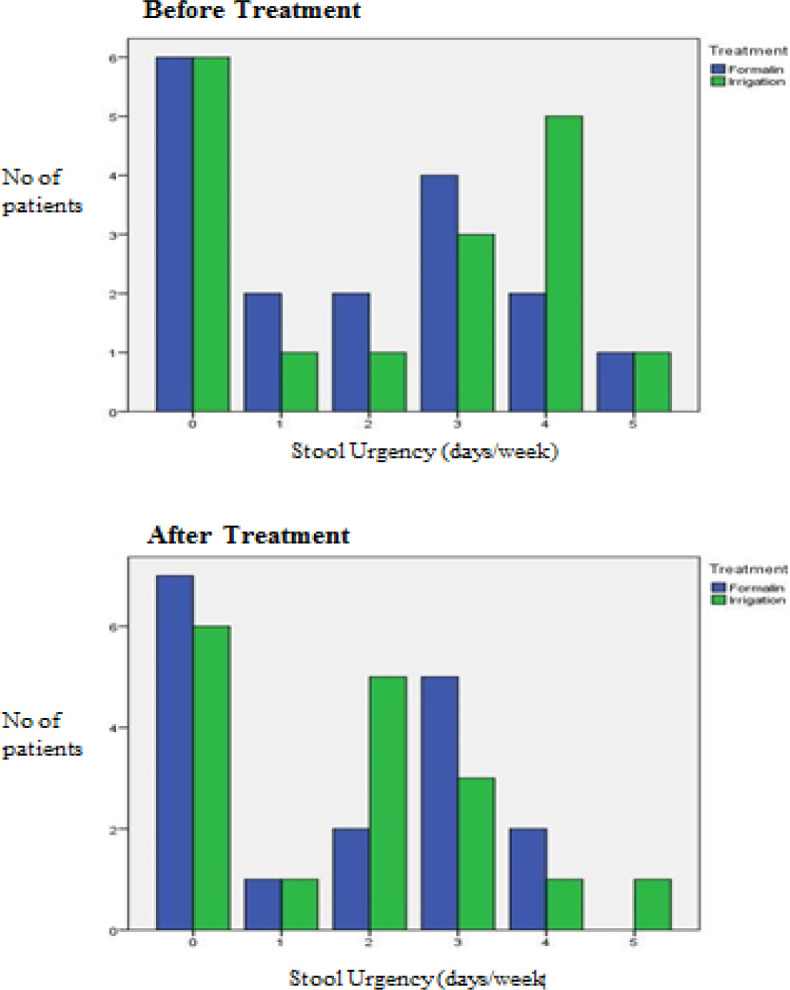
Stool Urgency before and after Treatment

**Figure 7:**
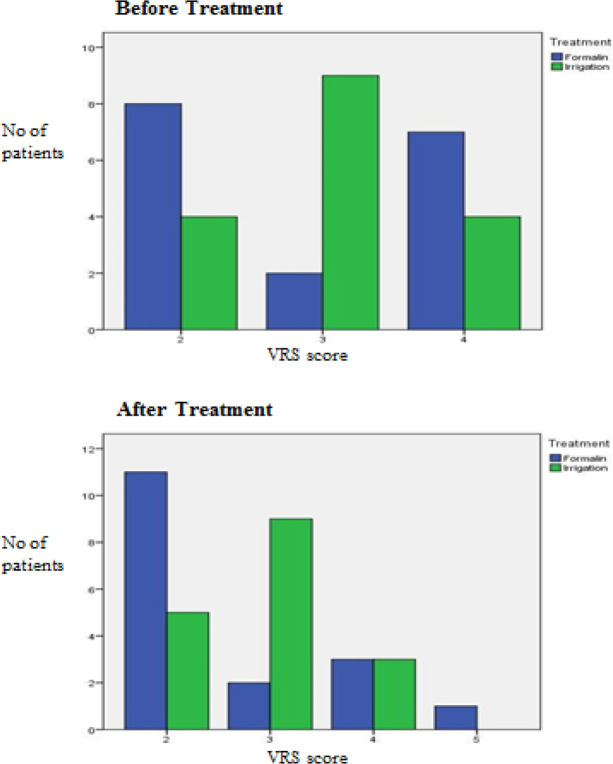
Vienna Rectoscopy Score (VRS) before and after Treatment

The results before and after treatment of both groups are shown in Figures 2 to 7. This study showed that patients in the formalin group had improvements in per rectal bleeding, diarrhoea, stool frequency and VRS score (Table 2). However, only improvement in per rectal bleeding was statistically significant. In the irrigation group, improvements were noted in per rectal bleeding, diarrhoea, tenesmus, stool frequency, stool urgency and VRS score (Table 3). Out of these, improvement in diarrhoea and tenesmus were significant. When we compared between the two groups, the only significant improvement seen was tenesmus. There was no significant difference in the other parameters (Table 4). One patient from each group experienced worsened per rectal bleeding after the treatment. Both were then given the crossover treatment. 

**Table 1:** Patients with Hemorrhagic Radiation Proctitis (N=34)

Parameters	Overall	Formalin	Irrigation	p
Age	59 (37 - 78)	62 (37 - 78)	56 (40 - 76)	0.132
Race				
Chinese N(%)	18 (53)	10 (59)	8 (47)	
Dayak N(%)	11 (32)	3 (18)	8 (47)	
Malay N(%)	5 (15)	4 (24)	1 (6)	
Primary Cancer				
Cervical Cancer N(%)	18 (53)	8 (47)	10 (59)	
Uterine Cancer N(%)	15 (44)	8 (47)	7 (41)	
Vaginal Cancer N(%)	1 (3)	1 (6)	0 (0)	
Onset of bleeding (month)	11.8 (6 - 54)	12.6 (6 - 54)	10.9 (6 - 18)	0.562
Duration of bleeding (month)	14.5 (1 - 66)	15.7 (1 - 66)	13.4 (1 - 50)	0.722
Previous treatment				
APC and Steriod enema N(%)	7 (21)	4 (24)	3 (18)	
Formalin and Steriod enema N(%)	2 (6)	1 (6)	1 (6)	
Steriod enema N(%)	4 (12)	2 (12)	2 (12)	
No treatment N(%)	21 (62)	10 (59)	11 (65)	
Previous blood transfusion N(%)	3 (9)	1 (6)	2 (12)	
Complications				
Anorectal discomfort N(%)	15 (44)	15 (88)		
Lower abdominal pain N(%)	1 (3)		1 (6)	
Worsening of bleeding N(%)	2 (6)	1 (6)	1 (6)	

Data shown in mean (range) or person (%); p, independent t-test

**Table 2:** Results of Patients Who Underwent Formalin Therapy (N=17)

Parameters	Before treatment	After treatment	p
Bleeding (days/week)	3 (2 - 7)	3 (1 - 3)	0.003
Diarrhoea (days/week)	0 (0 - 2)	0 (0 - 0)	0.288
Tenesmus (days/week)	0 (0 - 1)	0 (0 - 1)	0.317
Stool frequency (times/day)	2 (1 - 3)	2 (1 - 2)	0.26
Stool urgency (days/week)	2 (0 - 3)	2 (0 - 3)	0.317
VRS	3 (2 - 4)	2 (2 - 3)	0.131

Data shown as median (interquartile range); p, Wilcoxon signed rank test

**Table 3:** Results of Patients Who Underwent Irrigation Therapy (N=17)

Parameters	Before treatment	After treatment	p
Bleeding (days/week)	3 (2 - 7)	2 (1 - 7)	0.061
Diarrhoea (days/week)	0 (0 - 2)	0 (0 - 0)	0.018
Tenesmus (days/week)	0 (0 - 2)	0 (0 - 0)	0.024
Stool frequency (times/day)	3 (2 - 4)	2 (2 - 3)	0.271
Stool urgency (days/week)	3 ( 0 - 4)	2 (0 - 3)	0.215
VRS	3 (3 - 3)	3 (2 - 3)	0.157

Data shown as median (interquartile range); p, Wilcoxon signed rank test

**Table 4:** Comparison between the Differences of Two Treatment Groups

Parameters	Formalin group	Irrigation group	p
Bleeding (days/week)	-1(-2 - 0)	0 (-1 - 0)	0.115
Diarrhoea (days/week)	0 (-1 - 0)	0 (-2 - 0)	0.278
Tenesmus (days/week)	0 (0 - 0)	0 (-2 - 0)	0.043
Stool frequency (times/day)	0 (0 - 0)	0 (-1 - 0)	0.894
Stool urgency (days/week)	0 (0 - 0)	0 (-1 - 0)	0.465
VRS	0 (0 - 0)	0 (0 - 0)	0.836

Data shown as median (interquartile range); Difference, difference of data between "After treatment" and "Before treatment"; p = Mann-Whitney U test

## Discussion

At the moment there is no gold standard treatment for hemorrhagic radiation proctitis. Hence, we are still constantly searching for the best treatment. Medical therapy is slow and has limited success. Likewise, hyperbaric oxygen therapy’s current evidence is limited with marked variability and this alternative is expensive. Surgery is associated with undesirable higher morbidity and always remains as the last option. Hence, the preferences of endoscopic therapy like APC and formalin application. However, these treatments have limitations due to the cost of equipments and require frequent visits to clinic or hospital. The new technique of colonic irrigation and oral antibiotics interestingly showed better results than formalin (Sahakitrungruang et al., 2012). Therefore, it is important to explore this new method to find the answer for a better treatment modality.

In our cohort of patients, formalin therapy was effective in improving per rectal bleeding concurring to previous studies. The irrigation and oral antibiotics improved diarrhoea and tenesmus. However, the improvement in bleeding was statistically insignificant. The severity of per rectal bleeding based on the number of bleeding days/week used in the initial study and our study may not fully represent the true severity of per rectal bleeding. For example, 12 (35.3%) of our patients scored 7 days/week per rectal bleeding and 7 (20.6%) still remained as such after completion of treatment (Figure 1). In actual fact, these patients had improvement of symptoms as they experienced less amount of per rectal bleeding despite still having same bleeding days/week. Hence, a proper bleeding score encompassing bleeding frequency and estimated amount would have depicted the true bleeding severity and yielded a better and significant result.

When compared to the initial study (Sahakitrungrunag et al., 2012), the irrigation group showed more significant results. Our series only showed significant results in diarrhoea and tenesmus reduction. The initial study also showed that irrigation was superior to formalin in reducing per rectal bleeding, stool urgency and diarrhoea where as ours showed that irrigation was better than formalin in improving tenesmus only. 

In our opinion, this was attributed to our recruitment of patients who had undergone previous treatment but still had significant per rectal bleeding. They amount to 38% of our total patients. However, all of them did not receive any treatment at least one month prior to the study. Contrarily, the authors in the initial study only recruited patients without prior treatment. Hence, to begin with, our group of patients generally had milder but more chronic symptoms. A good example was the median per rectal bleeding in our study was 3 days/week compared to 7 days/week in the initial study. Hence, it is comprehensible for the initial study to yield more significant results compared to ours. It is important to include such patients as they made up to about one third of total patients with hemorrhagic radiation proctitis in our setting. A significant number of them experience intractable per rectal bleeding despite treatment. These patients are generally hard to treat as they may be resistant to treatment. In view of these reasons, perhaps the 8 week treatment is not adequate to observe a significant treatment outcome. A longer treatment period might be needed. We also believed that we could have gotten a better result for irrigation technique based on better counseling and patient education. This is to ensure proper irrigation technique and good compliance. 

The limiting factor for our study is our small sample size. It would have benefited from a multicentre study with more patient recruitment to illustrate a better study outcome. Another challenging factor was to educate the patients undergoing irrigation to ensure compliance and proper technique. Moreover, we need to have a proper bleeding scoring system to better assess per rectal bleeding severity. 

Nevertheless, this new therapy as shown in initial and current studies did show benefit in treating hemorrhagic radiation proctitis. It is generally safe with minimal complications. It can be used as a new treatment option which is easily self-administered at home and at the patient’s convenient time. It can also be used as combination treatment with endoscopic therapy to enhance the treatment outcome on per rectal bleeding and other anorectal symptoms associated with chronic radiation proctitis. 

In conclusion, treating hemorrhagic radiation proctitis is challenging which often requires multimodality and repeated therapies. It is also a chronic condition with symptoms recurring after a period of symptoms control. Water irrigation and oral antibiotics have shown effective and promising results to treat this condition. Hence, it could be a new, safe and more convenient treatment modality and can be combined with other established therapy to improve treatment outcome for hemorrhagic radiation proctitis.
